# Health-Related Quality of Life, Depressive Symptoms, Anxiety, and Somatization Symptoms in Male and Female Patients with Chronic Tinnitus

**DOI:** 10.3390/jcm10132798

**Published:** 2021-06-25

**Authors:** Benjamin Boecking, Raphael Biehl, Petra Brueggemann, Birgit Mazurek

**Affiliations:** Tinnitus Center, Charité—Universitätsmedizin Berlin, Charitéplatz 1, 10117 Berlin, Germany; benjamin.boecking@charite.de (B.B.); raphael.biehl@charite.de (R.B.); petra.brueggemann@charite.de (P.B.)

**Keywords:** chronic tinnitus, gender, health-related quality of life, psychological case conceptualization

## Abstract

Objective: To investigate the joint impact of tinnitus-related distress (TRD), anxiety, depressive symptoms, and other somatization symptoms on health-related quality of life (HRQoL) in female vs. male patients with chronic tinnitus. Method: Three-hundred-and-fifty-two patients with chronic tinnitus completed audiological testing and a psychological assessment battery that comprised—among other measures—German versions of the Tinnitus Questionnaire, Hospital Anxiety and Depression Scale, Somatic Symptom Scale-8, and Health-Related Quality of Life scale. Descriptive analyses examined associations as well as within- and between-gender differences of the measured variables. Gender-specific serial indirect effects analyses aimed to explain the impact of TRD on HRQoL through psychological processes, notably anxiety, depressive symptoms, and somatization symptoms. Results: Both female and male patients yielded lower mental than physical HRQoL and negative associations between the measured psychological variables and HRQoL. Compared to male patients, female patients reported higher levels of tinnitus-related- and wider psychological distress, other somatization symptoms (e.g., headaches), and impairments in mental and physical HRQoL. For each gender, depressive symptoms, anxiety, and somatization symptoms fully mediated the effect of TRD on mental and physical HRQoL. A double-dissociation revealed an interaction of somatization symptoms and depression on the TRD-HRQoL association in women, and of somatization symptoms and anxiety in men. Conclusions: In patients with chronic tinnitus, psychological constructs account for reported impairments in both mental and physical HRQoL. To improve patients’ HRQoL, treatment conceptualizations should consider gender-specific psychological expressions of low mood or anxiety.

## 1. Introduction

Chronic tinnitus denotes a long-lasting (>3 months) auditory percept without sound stimulation. Tinnitus prevalence rates range from 5 to 43%, and the prevalence of reported bothersome tinnitus from 3 to 31% [1].

While acute tinnitus can occur in the context of numerous otological or neurological conditions, little is known about factors that facilitate chronicity [2,3]. Phenomenologically, however, chronic tinnitus-related distress (TRD) is often expressed alongside—or potentially in lieu of—psychological or psychosomatic phenomena including depressive symptoms [4,5], anxiety [6,7,8], or other somatization symptoms that may or may not occur in the context of identifiable medical factors such as vertigo, sweats, blurred vision, headaches, periods of weakness, pain, nausea, or shortness of breath [9,10]. Thus, while the tinnitus sound might originate from audiological or somatosensory factors, chronicity appears to develop along a cognitive-emotional trajectory [11] which likely involves complex psychological vulnerability–stress interactions [12].

Numerous studies [13,14,15,16,17] have demonstrated effects of psychological or medical afflictions on, respectively, mental or physical health-related Quality of Life (HRQoL), i.e., perceived impairments in individuals’ health status caused by emotional or bodily factors [18,19]. Tinnitus-related distress [20,21,22], anxiety [23], depressive symptoms [24,25], and somatization symptoms [26,27,28] have all been reported to have a negative impact on patients’ health-related quality of life (HRQoL). Perhaps unsurprisingly, somatoform conditions or indeed any somatic condition that is experienced as psychologically burdensome may affect both indices. 

Previous studies demonstrated negative associations between both TRD and depressive symptoms and HRQoL. For example, Zeman et al. [29] investigated 1274 tinnitus patients from Argentina, Belgium, Brazil, Germany, Switzerland, and the USA. The authors reported strong and consistent relationships between self-reported tinnitus burden, depressive symptoms, and impaired HRQoL indices and argued that tinnitus severity could even be used as a proxy for depressive symptoms given how strongly these constructs overlapped conceptually. Weidt et al. [30] replicated these findings in a study with 208 tinnitus outpatients: while self-reported TRD explained significant variance of depressive symptoms and HRQoL, audiometric features were not associated with either variable. Moreover, studies using categorical diagnostic conceptualizations of tinnitus (as “index disorder”) and psychological “comorbidities” reported high co-occurrence rates of chronic tinnitus and anxiety, depressive symptoms, and somatization symptoms [31,32,33].

Broadening the picture, gender-specific differences have been emphasized for conceptualizations of patients’ presenting difficulties, treatment plans, and mental and medical healthcare pathways [34,35,36,37]. Overall, available evidence suggests that women tend to report higher HRQoL impairments in the context of psychological [38,39,40] or medical difficulties [41,42]. For chronic tinnitus, similar patterns emerged with women describing higher levels of emotional tension, psychological distress, and functional impairment [43,44,45,46], although these findings remain inconclusive [47,48]. To date, investigations on gender-specific differences in HRQoL for patients with chronic tinnitus are sparse. No study has yet investigated [a] the joint impact of TRD, anxiety, depressive symptoms and concurrent somatization symptoms on HRQoL and [b] whether such effects may differ for female vs. male patients with chronic tinnitus. We thus examined the following hypotheses:

1. Both female and male patients with chronic tinnitus report impairments in mental- and physical HRQoL.
2. Compared to male patients with chronic tinnitus, female patients report higher levels of TRD, anxiety, depressive symptoms, somatization symptoms, and lower HRQoL.
3. Within each gender, HRQoL is negatively influenced by TRD, anxiety, depressive symptoms, and somatization symptoms.
4. Within each gender, the impact of TRD on HRQoL is completely mediated by psychological constructs (anxiety, depressive symptoms and somatization symptoms).
5. [Exploratory] The observed indirect effects differ between female and male patients.

## 2. Method

### Procedure

The current study included *N* = 352 patients with chronic tinnitus who self-referred to the Tinnitus Center at Charité–Universitätsmedizin Berlin between January and December 2019. At an initial visit, patients completed audiological testing and, at a subsequent visit, a questionnaire battery that featured, among other measures, German versions of the [1] Tinnitus Questionnaire (TQ) measuring tinnitus-related distress, [2] Hospital Anxiety and Depression Scale (HADS) measuring anxiety and depressive symptoms, [3] Somatic Symptom Scale-8 (SSS-8)–measuring the perceived burden of common somatic symptoms known to reflect possible somatization tendencies, and [4] Short-Form-Health Survey (SF-12) Version 2.0 measuring mental and physical health-related quality of life. Here, patients were further asked, if they subjectively experienced hearing impairment. All data were collected as part of the clinic’s routine diagnostic procedures approved by the ethics committee of Charité Universitätsmedizin Berlin (EA4/216/20). All participants gave written consent for the use of anonymized data for research purposes in accordance with the Declaration of Helsinki. 

## 3. Materials

### 3.1. Tinnitus Questionnaire—German Version (TQ)

Tinnitus-related distress was measured using the Tinnitus Questionnaire [49]. The self-report questionnaire consists of 52 items (3-point-scale: “disagree” = 0, “partly agree” = 1, “agree” = 2). The total score is calculated over 40 items two of which are counted twice. The total score ranges from zero to 84 points with higher scores indicating higher tinnitus-related distress. The test–retest reliability of the TQ total score is 0.94 [49]. The questionnaire has been validated in various languages and populations [49], and the scale’s internal consistency was excellent (α = 0.94) in the current sample. Previous studies demonstrated age- and gender-related differences in TQ scores with women (vs. men) and middle-aged patients (vs. older or younger patients) tending to report higher TRD [50,51]. 

### 3.2. Hospital Anxiety and Depression Scale–German Version (HADS)

Anxiety and depressive symptoms were measured using the Hospital Anxiety and Depression Scale (HADS; [52]). The scale is a 14-item screening instrument detecting anxiety (HADS-A) and depressive symptoms (HADS-D). Each item is rated on a 4-point scale with higher scores indicating higher symptom severity. The internal consistency for the HADS-A is α = 0.80 and for the HADS-D α = 0.81 [52]. In the current sample, internal consistency was acceptable and good, respectively (α_HADS-A_ = 0.78; α_HADS-D_ = 0.87). Regarding age and gender-related influences, Hinz & Brähler [53] demonstrated in a representative German sample of *N* = 4410 participants that women (vs. men) tended to report higher HADS-A scores and older (vs. younger) people higher HADS-D scores. Langvik et al. [54] also reported higher anxiety scores in women (vs. men) and, additionally, higher depressive symptom scores in men (vs. women). 

### 3.3. Somatic Symptom Scale-8–German Version (SSS-8)

Somatization symptoms were measured using the Somatic Symptom Scale-8 (SSS-8, [55]). The SSS-8 was originally developed as the shortened version of the Patient Health Questionnaire (PHQ-15) aiming to detect somatic symptom burden. The scale comprises eight items that measure subjective impairment related to non-specific physical symptom groups: stomach or bowel problems, back pain, pain in arms/legs/joints, headaches, chest pain/shortness of breath, dizziness, feeling tired/having low energy, trouble sleeping. All symptoms are typical for somatization symptoms that are found in “somatoform disorders” [56,57]; however, any of the symptoms may equally occur as part of medical symptom presentations. All items are rated on 5-point-scale: 0 = “not at all”, 1 = “a little bit”, 2 = “somewhat”, 3 = “quite a bit”, 4 = “very much”. The reliability of the SSS-8 is good α = 0.81 [55]. In the current sample, the scale’s internal consistency was good (α = 0.81). The SSS-8 is commonly used as an indicator for psychosomatic symptoms or somatoform afflictions [58,59,60]. In the present study, the SSS-8 was used as a screening tool for somatization symptoms. 

### 3.4. Short-Form Health Survey—German Version (SF-12)

The Short-Form Health Survey (SF-12) is a 12-item screening instrument that assesses health-related quality of life (HRQoL [61]). A mental component summary scale (MCS; including Vitality, Social-Functioning, Role-Emotional, Mental Health) measures mental HRQoL. It includes 6 items that are answered on a 5-point-scale with higher scores indicate higher mental quality of life. Analogously, a physical component summary scale (PCS; including Physical-Functioning, Role-Physical, Bodily Pain, and General Health) reflects an individual’s physical HRQoL. The PCS includes 2 items that are answered on a 3-point-scale and 4 items answered on a 5-point-Likert scale with higher scores indicating higher physical HRQoL. For both scales, raw scores are transformed to population-mean-adjusted T-Scores with a mean of 50 and a standard deviation of 10. Internal consistencies of the summary scales are reportedly good (PCS: α = 0.89; MCS: = 0.79; [61]). In the current sample, internal consistencies were good (α_PCS_ = 0.88; α_MCS_ = 0.89). Previous studies using this measure demonstrated reduced HRQoL in women (vs. men) and older (vs. younger) individuals [62].

## 4. Participants

A total of *N* = 352 patients with chronic tinnitus (54.3% female) completed audiological testing as well as the TQ, HADS, SSS-8, and SF-12 across two appointments as part of the clinic’s diagnostic psychosomatic intake procedures. All questionnaire measures were completed in the secure REDCap© application run on iOS Apple iPad© devices. On average, patients were 52.65 years old (*SD* = 11.37; range = 20–82 years; women: *M* = 53.32, *SD* = 11.13; men: *M* = 51.85, *SD* = 11.65; *t*(350) = −1.207; *p* > 0.05). On average, 80.50 days (*SD* = 108) passed between audiological testing and questionnaire completion.

## 5. Statistical Analyses

All analyses were conducted using IBM SPSS Statistics for Windows, Version 26. Statistical significance was set at *p* = 0.05. First, we computed descriptive statistics and correlation coefficients. Second, paired-samples *t*-tests investigated within-gender differences of anxiety vs. depression, and mental vs. physical HRQoL scores. Third, independent samples *t*-tests investigated between-gender differences for the obtained measures. Between-gender differences in correlation coefficients [63] and effect sizes *d* [64] were calculated using an online tool. Here, effect sizes are defined as *d* (0.01) = very small, *d* (0.20) = small, *d* (0.50) = medium, *d* (0.80) = large, *d* (1.20) = very large, and *d* (2.00) = huge [65]. Fourth, to explore interdependent effects between tinnitus-related distress, other psychological or psychosomatic phenomena (anxiety, depressive symptoms, and somatization symptoms), and HRQoL, serial multiple mediation analyses specified tinnitus-related distress as independent, anxiety as first-step mediating, depressive symptoms as second-step mediating, somatization symptoms as third-step mediating, and mental or physical HRQoL as dependent variables. The order of mediators was determined by both theoretical (e.g., overall higher co-occurrence rates of tinnitus-related distress and anxiety [vs. depression] in the literature) and empirical considerations (higher levels of anxiety than depressive symptoms within each gender in the present sample, see Table 4). Overall, four models (gender [male, female] × HRQoL [mental, physical]) were calculated using PROCESS [66]. In all analyses, age was included as covariate. We report *β* weights for the standardized total indirect effect. For these *β* weights, coefficients of >0.10 denote a small, >0.30 a medium, and >0.50 a large effect. In the results section, indirect effects are reported graphically (for an overview of numerical effects, see Supplementary Materials Figure S1 and Table S1).

## 6. Results

Table 1, Table 2, Table 3, Table 4 and Table 5 report sociodemographic variables (Table 1), hearing- and tinnitus-related variables (Table 2), Pearson correlation coefficients *r* (Table 3) for the investigated variables within each gender, within-gender comparisons of mood and HRQoL indices (Table 4), and between-gender differences (Table 5). While most participants were of un- or only slightly impaired hearing ability, a somewhat larger proportion (female patients: 64%; male patients: 55%) reported subjective perceptions of hearing impairment (“Is your hearing ability impaired?” [Yes; No]). The majority of patients reported binaural tinnitus perceptions. Both women and men reported higher levels of anxiety vs. depression and lower levels of mental vs. physical HRQoL. Compared to men, women showed higher levels of distress across all measures except for low mood, which was comparable for both genders. Most psychological variables intercorrelated highly. In addition, small significant correlations emerged between participants’ hearing ability and tinnitus-related distress as well as physical, but not mental HRQoL.

### 6.1. Serial Mediation Analyses

In order to explore possible interactions of psychological factors (anxiety, depressive symptoms, and other somatization symptoms) in explaining associations between TRD and mental or physical HRQoL, we computed four (gender [male, female] × HrQoL [mental, physical]) sets of mediation analyses. Here, we specified the TQ total score as independent, HADS-A, HADS-D, and SSS-8 scores as mediating, and mental or physical HRQoL as dependent variables (Hypotheses 3–5).

#### 6.1.1. Mental HRQoL in Female Patients with Chronic Tinnitus

**Figure 1:**
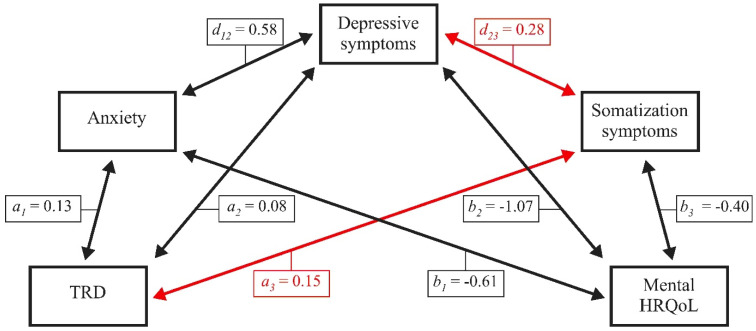
Anxiety, depressive symptoms, and somatization symptoms completely mediate the effect of TRD on mental HRQoL in female patients with chronic tinnitus. TRD = tinnitus-related distress; HRQoL = health-related quality of life. Numbers indicate unstandardized multiple regression coefficients. Red lines indicate effects that are significant in female patients only.

Figure 1 illustrates effects of anxiety, depressive symptoms, and somatization symptoms on the association between TRD and mental HRQoL in female patients with chronic tinnitus. Results indicate a statistically significant total effect (*β* = −0.34, *SE* [standard error] = 0.03, *LLCI* [lower limit confidence interval] = −0.41, *ULCI* [upper limit confidence interval] = −0.27), that is completely mediated by the three included psychological factors as shown below (total indirect effect, *β* = −0.33, *SE* = 0.03, *LLCI* = −0.39, *ULCI* = −0.26; standardized total indirect effect, *β* = −0.52, *SE* = 0.05, *LLCI* = −0.61, *ULCI* = −0.43).

#### 6.1.2. Physical HRQoL in Female Patients with Chronic Tinnitus

**Figure 2:**
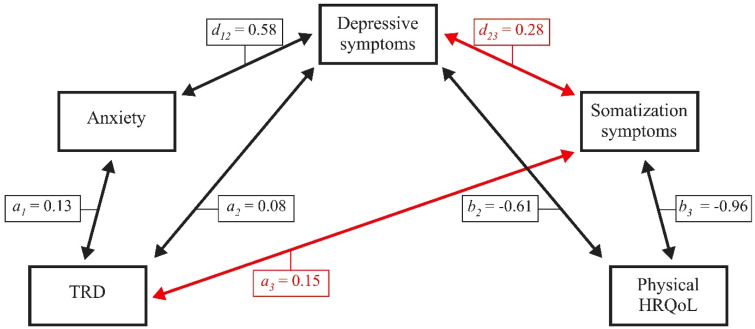
Anxiety, depressive symptoms, and somatization symptoms completely mediate the effect of TRD on physical HRQoL in female patients with chronic tinnitus. TRD = tinnitus-related distress; HRQoL = health-related quality of life. Numbers indicate unstandardized multiple regression coefficients. Red lines indicate effects that are significant in female patients only.

Figure 2 illustrates indirect effects for the association between TRD and physical HRQoL in female patients with chronic tinnitus. Results indicate a statistically significant total effect (*β* = −0.33, *SE* = 0.03, *LLCI* = −0.39, *ULCI* = −0.27) that is completely mediated by the three psychological factors (total indirect effect, *β* = −0.27, *SE* = 0.03, *LLCI* = –0.33, *ULCI* = −0.22; standardized total indirect effect, *β* = −0.46, *SE* = 0.04, *LLCI* = −0.54, *ULCI* = −0.38).

#### 6.1.3. Mental HRQoL in Male Patients with Chronic Tinnitus

**Figure 3:**
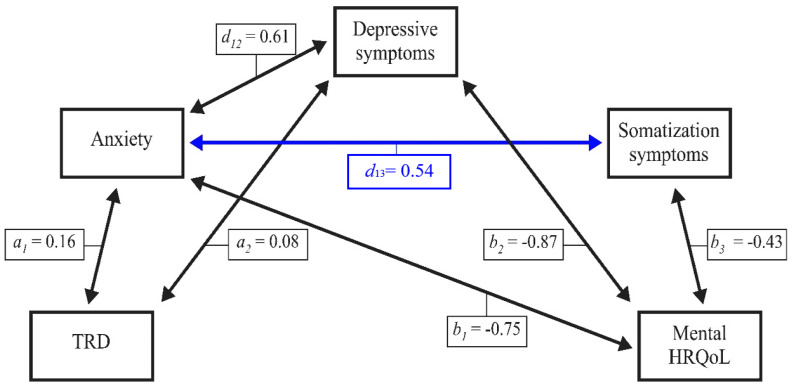
Anxiety, depressive symptoms, and somatization symptoms completely mediate the effect of TRD on mental HRQoL in male patients with chronic tinnitus. TRD = tinnitus-related distress; HRQoL = health-related quality of life. Numbers indicate unstandardized multiple regression coefficients. Blue lines indicate effects that are significant in male patients only.

Figure 3 illustrates indirect effects for the association between TRD and mental HRQoL in male patients with chronic tinnitus. Results indicate a statistically significant total effect (*β* = −0.39, *SE* = 0.04, *LLCI* = −0.47, *ULCI* = −0.31) that is completely mediated by the three psychological factors as shown below (total indirect effect, *β* = −0.36, *SE* = 0.03, *LLCI* = −0.42, *ULCI* = −0.29; standardized total indirect effect, *β* = −0.52, *SE* = 0.05, *LLCI* = −0.61, *ULCI* = −0.43).

#### 6.1.4. Physical HRQoL in Male Patients with Chronic Tinnitus

**Figure 4:**
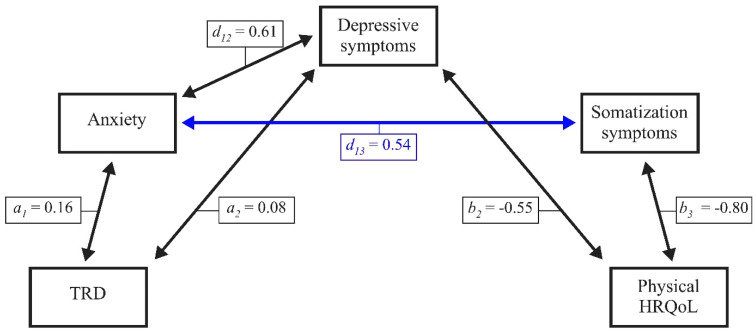
Anxiety, depressive symptoms, and somatization symptoms completely mediate the effect of TRD on physical HRQoL in male patients with chronic tinnitus. TRD = tinnitus-related distress; HRQoL = health-related quality of life. Numbers indicate unstandardized multiple regression coefficients. Blue lines indicate effects that are significant in male patients only.

Last, Figure 4 illustrates indirect effects for the association between TRD and physical HRQoL in male patients with chronic tinnitus. Again, results indicate a statistically significant total effect (*β* = –0.24, *SE* = 0.04, *LLCI* = –0.17, *ULCI* = –0.03) that is completely mediated by the three psychological factors as shown below (total indirect effect, *β* = –0.28, *SE* = 0.03, *LLCI* = –0.35, *ULCI* = –0.21; standardized total indirect effect, *β* = –0.48, *SE* = 0.05, *LLCI* = –0.58, *ULCI* = –0.37).

In both female and male patients with chronic tinnitus, (1) tinnitus-related distress was negatively associated with patients’ mental and physical HRQoL; (2) anxiety, depressive symptoms, and other somatization symptoms accounted for these associations; (3) TRD, anxiety, and depressive symptoms were associated with each other; (4) mental HRQoL was associated with anxiety, depressive symptoms, and somatization symptoms, and (5) physical HRQoL with depressive and somatization symptoms, but not anxiety.

In female patients, (6) somatization symptoms interacted with TRD and depressive symptoms, but not anxiety in explaining the effect of TRD on mental (Figure 1) or physical HRQoL (Figure 2). By contrast, in male patients, (7) somatization symptoms interacted with anxiety, but not TRD and/or depressive symptoms in explaining the effect of TRD on mental (Figure 3) or physical (Figure 4) HRQoL. 

## 7. Discussion

The present study investigated joint effects of tinnitus-related distress (TRD), anxiety, depressive symptoms, and other, non-tinnitus-related somatization symptoms on mental or physical health-related quality of life (HRQoL) in female and male patients with chronic tinnitus.

**Hypothesis** **1.** *Both female and male patients with chronic tinnitus report psychological distress alongside impairments in mental and physical HRQoL.*

Although some symptom variation was apparent, both women and men reported medium-to-high levels of TRD, mildly elevated psychological distress, and medium-to-high somatization symptom levels against the background of mild impairments in mental and physical HRQoL. The measured psychological constructs were highly correlated. In addition, small correlations emerged between patients’ hearing ability and tinnitus-related distress as well as physical, but not mental HRQoL. Comparing symptoms within each gender, both female and male patients showed higher levels of anxiety than depressive symptoms, and higher impairments in mental than physical HRQoL. Consequently, while patients with chronic tinnitus reported impairments across both HRQoL domains, chronic tinnitus appeared to primarily affect or be reflected in patients’ mental HRQoL. Interestingly, although patients’ hearing ability appeared to exert a small influence on tinnitus-related distress and physical HRQoL, the majority of patients in our sample showed no or only slight hearing impairment. Rather, patients’ physical HRQoL impairments appeared attributable to psychological factors or, respectively, psychosomatic interactions. These findings highlight the central importance of psychological constructs (1) for perceived impairments in both mental and physical HRQoL in patients with chronic tinnitus similar to HRQoL patterns reported in other somatoform conditions [16], [67] and (2) as important treatment targets. Our results support recommendations for psychologically informed interventions as first-line treatments for patients with chronic tinnitus [68,69,70,71].

**Hypothesis** **2.** *Compared to male patients with chronic tinnitus, female patients report higher levels of TRD, anxiety, depressive symptoms, and somatization symptoms, and lower HRQoL.*

Compared to male patients with chronic tinnitus, female patients reported higher levels of TRD, anxiety, and somatization symptoms, and lower levels of HRQoL. This finding is in keeping with some previous studies that emphasized higher levels of tinnitus-related distress [51] as well as psychological epiphenomena [50,51,72] in women with chronic tinnitus, although the literature is mixed to this regard (e.g., [73]). Prevalence studies focusing on psychological distress in general have frequently reported higher rates of depressive symptoms, anxiety, and somatoform symptom presentations in women than men [74,75,76,77,78]. To this regard, different explanatory perspectives have been adopted including higher social burden in women due to social discrimination or economic inequality [79] or distinct gender-related biopsychological phenotypes [80,81]. Conversely, the findings might reflect lower levels of emotional awareness or underreporting of emotional distress by men—potentially based on masculine gender norms and associated stigmatization concerns [82,83,84,85,86,87,88]. The present data do not allow for an investigation of these hypotheses; however, future research ought to consider gender-associated sociological and psychological factors in explaining wider distress presentations involving chronic tinnitus symptomatology.

**Hypothesis** **3.** *In each gender, HRQoL is negatively influenced by TRD, anxiety, depressive symptoms, and somatization symptoms.*

**Hypothesis** **4.** *The impact of TRD on HRQoL is completely mediated by these psychological constructs.*

In both female and male patients with chronic tinnitus, (1) tinnitus-related distress was negatively associated with patients’ mental and physical HRQoL; (2) anxiety, depressive symptoms, and other somatization symptoms completely mediated these associations; (3) TRD, anxiety, and depressive symptoms were associated with each other; (4) mental HRQoL was associated with anxiety, depressive symptoms, and somatization symptoms; and physical HRQoL with depressive and somatization symptoms, but not anxiety. Once more, this finding highlights the key role of psychological factors for tinnitus-related distress and its relation to impairments in patients’ HRQoL. Interestingly, anxiety, depressive symptoms, and somatization tendencies fully accounted for observed associations between tinnitus-related distress and HRQoL any longer. This finding highlights the importance of considering construct overlaps as well as conceptualizing tinnitus-related distress within broader psychological contexts. The finding lends further support to the argument that tinnitus-related distress and other somatic expressions of emotional distress (e.g., affective pain experiences) are attributable to psychological influences [89] whose treatment may jointly benefit different somatoform symptom clusters [90] and thereby also benefit patients’ mental and physical HRQoL. Our research suggests a psychotherapeutic focus on emotional phenomena linked to emotional internalization (anxiety, depression) or somatization tendencies [91,92,93]. Psychological therapies that focus on emotion regulation strategies may hence be beneficial for patients with chronic tinnitus [94,95] although research to date primarily applied traditional, disorder-specific cognitive-behavioural approaches [96].

**Hypothesis** **5.** *[Exploratory] Observed indirect effects differ between female and male patients.*

The interactional patterns of psychological factors that explain the association between tinnitus-related distress and impairments in HRQoL, the present study provided initial evidence for gender-specific differences. In female patients, (1) depressive symptoms were positively associated with both anxiety and somatization symptoms, (2) depressive symptoms appeared to account for the association between anxiety and somatization symptoms, and (3) somatization symptoms interacted with TRD and depressive symptoms, but not anxiety in explaining the effect of TRD on mental or physical HRQoL. In male patients, anxiety was positively associated with somatization symptoms, which were, however, not directly influenced by depressive symptoms. Somatization symptoms interacted with anxiety, but not TRD and/or depressive symptoms in explaining the effect of TRD on mental or physical HRQoL. These findings lend further support to a key role of depressive symptoms in the experience of tinnitus-related and -associated distress patterns [4], [97,98]—particularly in female patients with chronic tinnitus. Anxiety, by contrast, appears to emerge as a key treatment target in men with somatization symptoms. The reasons for these gender differences remain speculative at this point; however, they should be further investigated in psychotherapy research that highlights the importance of idiosyncratic case conceptualizations [99].

## 8. Limitations

The current study has several limitations: Most importantly, the interpretation and generalization of the data is limited by the cross-sectional nature of the data and the absence of a control group. Cross-sectional mediation analyses do not allow for causal inferences, yet suggest possible networks of intervariable associations that should be investigated in future longitudinal or experimental studies. Clinically, the applied questionnaires provide rather superficial accounts of psychological experiences involved in tinnitus-related distress. While they were selected due to their wide usage and limited response burden [100], developing more nuanced clinical pictures is clearly necessary. In this context, vulnerability stress or psychological case conceptualization frameworks might be helpful to identify common and specific influencing factors among the often-heterogeneous presentations of tinnitus-related and wider psychological distress [101], [102]. Moreover, although our somatization measure likely captures functional somatization phenomena, underlying medical factors cannot be ruled out and might impact upon individuals’ physical and mental HRQoL as well as psychological distress measures in their own right. Nonetheless, the results of the mediation analyses highlight the importance of psychological phenomena for patients’ physical as well as mental HRQoL impairments in the context of un- or only slightly impaired hearing ability.

## 9. Conclusions

The present study is the first to investigate gender differences in chronic tinnitus patients’ HRQoL regarding possible influences of tinnitus-related distress, anxiety, depressive symptoms, and other somatization phenomena. Psychological variables were observed to fully account for associations between tinnitus-related distress and impairments in mental and physical HRQoL. In terms of broader somatization tendencies and reported HRQoL impairments, gender-specific analyses revealed central roles of depressive symptoms for women and anxiety symptoms for men. In order to improve patients’ quality of life and tinnitus-related distress, these factors are to be paid particular attention within a wider picture of interconnected psychological phenomena. Future studies should investigate idiosyncratic transdiagnostic experience- (not symptom-) focused psychological phenomena such as “experiential avoidance” that have been shown to inform both anxiety-, depression-, and somatization-related phenomena (e.g., [103]). While psychological literature has long focused on gender-specific effects in the onset, maintenance, and treatment of emotional distress, such research ought to be broadened into the tinnitus field. Clinically, psychological case conceptualizations and treatment plans should consider the emerging empirical knowledge on gender-specific contributors to emotional distress in order to prevent chronicity, or to benefit patients’ HRQoL.

## Figures and Tables

**Table 1:** Sociodemographic variables.

Variable	Female		Male	
*n*	191		161	
	*M*	*SD*	*M*	*SD*
Age	53.32	11.13	51.85	11.65
	*n*	%	*n*	%
**Degree**				
None	2	1.0	2	1.2
School	59	30.9	56	34.8
Apprenticeship	42	22.0	25	17.4
Polytechnic degree	30	15.7	18	11.2
University degree	58	30.4	57	35.4
**Nationality**				
German	174	91.1	146	90.7
Other	17	8.9	15	9.3
**Relationship Status**				
Single	37	19.4	35	21.7
Married/Partner	114	59.7	112	69.6
Divorced	29	15.2	13	8.1
Widowed	11	5.8	1	0.6
**Work Status**				
Employed	133	69.6	116	72.0
Unemployed	58	30.4	45	28.0

Notes: *χ*²-tests indicated no between-gender differences for degree, nationality, and work status, yet a significant between-gender difference for relationship status with females (vs. males) being less frequently married and more frequently divorced or widowed, *χ*²(1) = 18.19, *p* = 0.00, *φ* = 0.23.

**Table 2:** Hearing- and tinnitus-related variables.

Variable	Female		Male	
*n*	191		161	
	*M*	*SD*	*M*	*SD*
PTA right	22.77	12.77	23.42	15.79
PTA left	22.88	14.10	23.88	13.62
	*n*	%	*n*	%
**Hearing impairment_right**				
None	141	73.8	118	73.3
Slight	39	20.4	27	16.8
Moderate	9	4.7	8	5.0
Severe	1	0.5	3	1.9
Profound	1	0.5	1	0.6
**Hearing impairment_left**				
None	141	73.8	112	69.9
Slight	38	19.9	33	20.5
Moderate	7	3.7	12	7.5
Severe	2	1.0	1	0.6
Profound	1	0.5	0	0
**Subjectively perceived hearing impairment**				
Yes	122	63.9	88	54.7
No	69	36.1	73	45.3
**Duration of tinnitus**				
<½ year	21	11.0	16	9.9
½–1 year	46	24.1	30	18.6
1–2 years	23	12.0	19	11.8
2–4 years	35	18.4	17	10.6
>4 years	66	34.6	79	49.1
**Localisation**				
left	36	18.8	20	12.4
right	35	18.3	30	18.6
binaural	113	34.6	106	65.8

Notes: PTA = Average Pure Tone Audiometry threshold (dB) across 0.25, 0.5, 1, 1.5, 2, 3, 4, 6, 8, 10 kHz. *χ*²-tests indicated no between-gender differences for (subjectively perceived) hearing impairment, tinnitus duration and tinnitus localization. Similarly, there were no between-gender PTA-related differences (*t*[345] = 0.42 [right] and 0.67 [left]; *p* > 0.05).

**Table 3:** Significant Pearson correlation coefficients *r* for the obtained measures. All coefficients are comparable between genders except where otherwise indicated.

	PTA Right		PTA Left		TQ		HADS-A		HADS-D		PCS		MCS	
	F	M	F	M	F	M	F	M	F	M	F	M	F	M
PTA left	0.650***	0.711***												
TQ	0.242**	0.216**	0.331***	0.230**										
HADS-A					0.547***	0.596***								
HADS-D	0.182*^,†^			0.175*^,†^	0.572***	0.619***	0.660***	0.719***						
PCS	−0.272***		−0.318**	−0.164*	−0.585***^,†^	−0.425***^,†^	−0.470***	−0.497***	−0.608***	−0.546***				
MCS					−0.564***	−0.585***	−0.660***	−0.746***	−0.750***	−0.744***	0.675***	0.627***		
SSS-8			0.164*		0.596***	0.474***	0.469***	0.544***	0.518***	0.498***	−0.756***	−0.678***	−0.596***	−0.609***

Notes: **^†^** Correlation coefficients differ between genders (*r*_TQ_PCS_ *z* = 2.004; *p* = 0.023; *r*_PTA_HADS-D_ *z* = 1.672; *p* = 0.047). * *p* < 0.05; ** *p* < 0.01; *** *p* < 0.001. F = Female; HADS-A = Hospital Anxiety and Depression Scale-Anxiety subscale; HADS-D = Depression subscale; M = Male; MCS = Short-Form Health Survey mental component summary scale; PCS = Short-Form Health Survey physical component summary scale; PTA = Pure Tone Average; SSS-8 = Somatic Symptom Scale-8; TQ = Tinnitus Questionnaire.

**Table 4:** Within-gender differences for anxiety vs. depression, and mental vs. physical HRQoL (paired-samples *t*-tests).

	**HADS-A**		**HADS-D**				
	*M*	*SD*	*M*	*SD*	*df*	*t*	*d*
Female	8.87	3.81	7.21	4.42	190	6.67 **	0.43
Male	7.40	4.15	6.50	4.56	160	3.48 ***	0.21
	**MCS**		**PCS**				
	*M*	*SD*	*M*	*SD*	*df*	*t*	*d*
Female	36.43	10.37	39.87	9.88	190	−5.68 ***	0.36
Male	39.03	10.81	43.06	9.23	160	−5.67 ***	0.39

Notes: ** *p* < 0.01; *** *p* < 0.001; *d* = *Cohen’s d* (0.20 = small; 0.50 = medium). *M* = mean, *SD* = standard deviation, *df* = degrees of freedom, HADS-A = Hospital Anxiety and Depression Scale-Anxiety subscale; HADS-D = Depression subscale; MCS = Short-Form Health Survey mental component summary scale; PCS = Short-Form Health Survey physical component summary scale; PTA = Pure Tone Average; SSS-8 = Somatic Symptom Scale-8; TQ = Tinnitus Questionnaire.

**Table 5:** Between-gender differences for the obtained measures (independent-samples *t*-tests).

	Women		Men			
	*M*	*SD*	*M*	*SD*	*t(350)*	*d*
TQ	42.46	16.22	37.01	15.92	−3.17 **	−0.34
HADS-A	8.87	3.80	7.40	4.15	−3.44 **	−0.37
HADS-D	7.21	4.42	6.50	4.56	−1.48	
SSS-8	12.96	5.79	10.09	6.05	3.68 ***	−0.49
MCS	36.09	10.31	39.26	10.95	2.78 **	0.30
PCS	39.44	9.91	43.23	9.35	−4.52 ***	0.40

Notes: ** *p* < 0.01; *** *p* < 0.001; *d* = *Cohen’s d* (0.20 = small; 0.50 = medium). *M* = mean, *SD* = standard deviation, HADS-A = Hospital Anxiety and Depression Scale-Anxiety subscale; HADS-D = Depression subscale; MCS = Short-Form-Health Survey mental component summary scale; PCS = Short-Form-Health Survey physical component summary scale; PTA = Pure Tone Average; SSS-8 = Somatic Symptom Scale-8; TQ = Tinnitus Questionnaire.

## Supplementary Materials

The following are available online at https://www.mdpi.com/article/10.3390/jcm10132798/s1, Figure S1: Pathway specifications for the present analyses, Table S1: Numerical effects for the serial multiple mediation analyses.
