# Supplementary material for: Health-Related Quality of Life, Depressive Symptoms, Anxiety, and Somatization Symptoms in Male and Female Patients with Chronic Tinnitus
Source: J Clin Med. 2021 Jun 25;10(13):2798. doi: 10.3390/jcm10132798 (PMC8267833; doi:10.3390/jcm10132798)
Supplement: Supplementary file 1 [file jcm-10-02798-s001.zip › jcm-1235333-supplementary.pdf]

Figure S1: Pathway specifications for the present analyses.

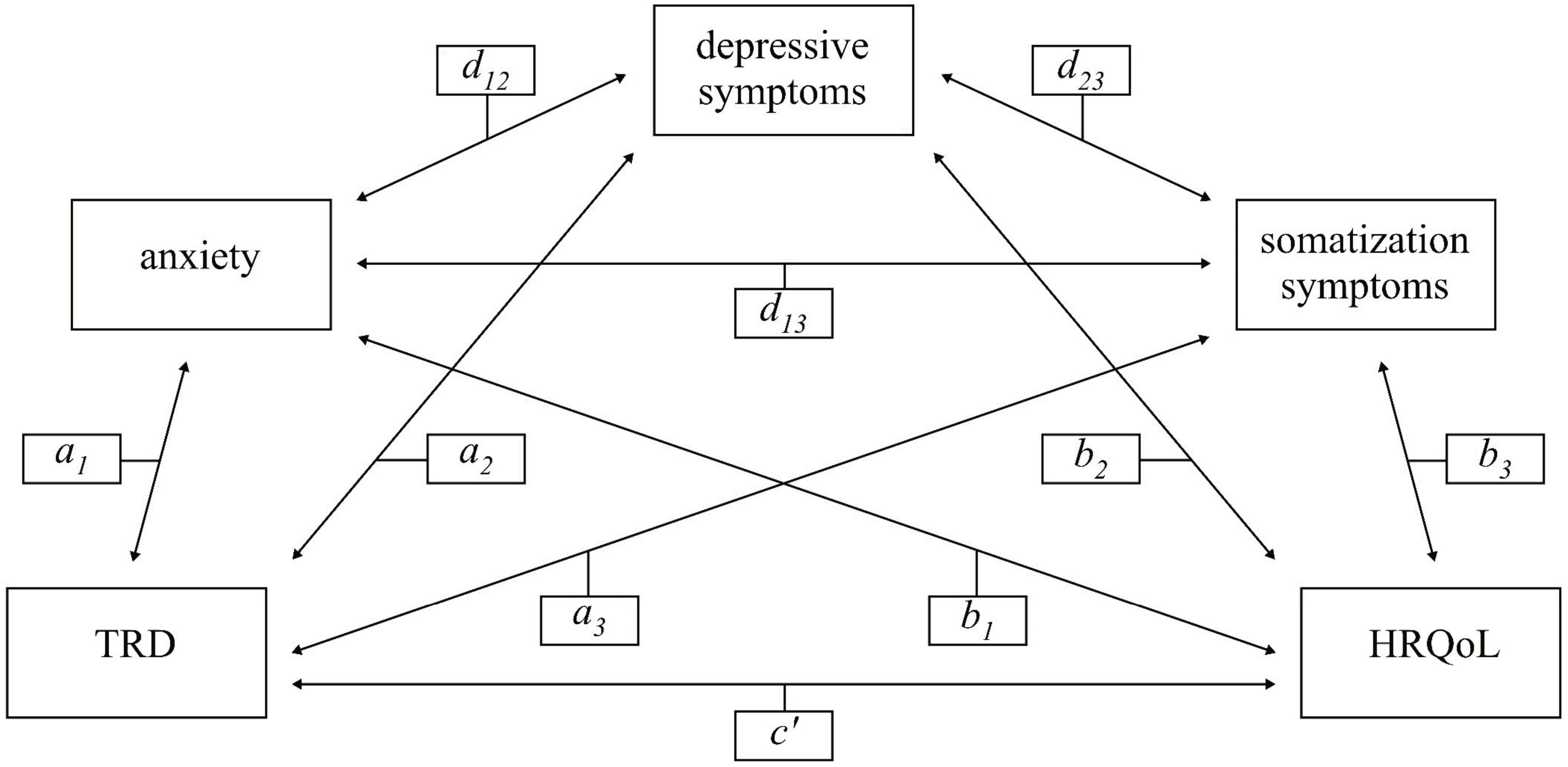


Details and coefficients for the mediation analyses are outlined featuring coefficients *a* (effects of the independent variables on the mediators), *b* (effect of the mediators on the dependent variable), *c* (total effect of the independent variable on the dependent variable), *c'* (the direct effect; i.e. the total effect adjusted for *ab*), *d* (effects between the mediators) and the total indirect effect that is tested for significance using a *bootstrapping* approach with 95% confidence intervals.

Table S1: Numerical effects for the serial multiple mediation analyses.

| | $a_1$ | se | llci | ulci | $a_2$ | se | llci | ulci | $a_3$ | se | llci | ulci | $b_1$ | se | llci | ulci | $b_2$ | se | llci | ulci |
|---|---|---|---|---|---|---|---|---|---|---|---|---|---|---|---|---|---|---|---|---|
| 1 | .13 | .01 | .10 | .16 | .08 | .02 | .05 | .11 | .15 | .03 | .10 | .20 | -.61 | .16 | -.94 | -.29 | -1.07 | .15 | -1.36 | -.78 |
| 2 | .13 | .01 | .10 | .16 | .08 | .02 | .05 | .11 | .15 | .03 | .10 | .20 | -.02 | .15 | -.31 | .27 | -.61 | .13 | -.87 | -.35 |
| 3 | .16 | .02 | .13 | .19 | .08 | .02 | .04 | .12 | .06 | .03 | -.01 | .13 | -.74 | .19 | -1.12 | -.37 | -.87 | .16 | -1.19 | -.55 |
| 4 | .16 | .02 | .13 | .19 | .08 | .02 | .04 | .12 | .06 | .03 | -.01 | .13 | -.18 | .20 | -.58 | .21 | -.55 | .17 | -.89 | -.21 |

| | $b_3$ | se | llci | ulci | $c$ | se | llci | ulci | $c'$ | se | llci | ulci | $d_{12}$ | se | llci | ulci | $d_{13}$ | se | llci | ulci | $d_{23}$ | se | llci | ulci |
|---|---|---|---|---|---|---|---|---|---|---|---|---|---|---|---|---|---|---|---|---|---|---|---|---|
| 1 | -.40 | .10 | -.60 | -.20 | -.36 | .04 | -.44 | -.29 | -.03 | .04 | -.10 | .05 | .58 | .07 | .44 | .72 | .15 | .12 | -.08 | .39 | .28 | .10 | .07 | .48 |
| 2 | -.96 | .09 | -1.14 | -.78 | -.34 | .04 | -.41 | -.27 | -.04 | .03 | -.11 | .03 | .58 | .07 | .44 | .72 | .15 | .12 | -.08 | .39 | .28 | .10 | .07 | .48 |
| 3 | -.43 | .10 | -.63 | -.23 | -.41 | .04 | -.50 | -.33 | -.06 | .04 | -.14 | .02 | .61 | .07 | .46 | .76 | .53 | .15 | .25 | .83 | .18 | .13 | -.07 | .44 |
| 4 | -.80 | .10 | -1.00 | -.59 | -.24 | .04 | -.33 | -.16 | .03 | .04 | -.06 | .11 | .61 | .07 | .46 | .76 | .53 | .15 | .25 | .83 | .18 | .13 | -.07 | .44 |

1 = model for Mental HRQoL in Female Patients with Chronic Tinnitus, 2 = model for Physical HRQoL in Female Patients with Chronic Tinnitus, 3 = model for Mental HRQoL in Male Patients with Chronic Tinnitus, 4 = model for Mental HRQoL in Male Patients with Chronic Tinnitus, se = standard error, llci = lower level confidence interval, ulci = upper level confidence interval
